# Tribological and Antimicrobial Properties of Two-Component Self-Assembled Monolayers Deposited on Ti-Incorporated Carbon Coatings

**DOI:** 10.3390/ma17020422

**Published:** 2024-01-14

**Authors:** Michał Cichomski, Natalia Wrońska, Mariusz Dudek, Anna Jędrzejczak, Katarzyna Lisowska

**Affiliations:** 1 Department of Materials Technology and Chemistry, Faculty of Chemistry, University of Lodz, Pomorska 163, 90-236 Lodz, Poland; 2 Department of Industrial Microbiology and Biotechnology, Faculty of Biology and Environmental Protection, University of Lodz, Banacha 12/16, 90-237 Lodz, Poland; natalia.wronska@biol.uni.lodz.pl (N.W.); katarzyna.lisowska@biol.uni.lodz.pl (K.L.); 3 Institute of Materials Science and Engineering, Lodz University of Technology, Stefanowskiego 1/15, 90-924 Lodz, Poland; mariusz.dudek@p.lodz.pl (M.D.); anna.jedrzejczak@p.lodz.pl (A.J.)

**Keywords:** self-assembled monolayers, silanes, Ti-incorporated carbon coatings, microtribology, antimicrobial activity

## Abstract

In this work, Ti-incorporated carbon coatings were used as substrates for modification with one- and two-component self-assembled monolayers of organosilane compounds using a polydimethylsiloxane (PDMS) stamp. This enabled the selective functionalization of surfaces with micrometric dimensions. The topography of the modified surfaces was defined using an atomic force microscope (AFM). The effectiveness of the modification was confirmed by measurements of the water contact angle and surface free energy using the Oss and Good method. Using a T-23 microtribometer with counterparts in the shape of balls that were made of steel, silicon nitride (Si_3_N_4_), and zirconium dioxide (ZrO_2_), the tribological properties of the obtained coatings were tested. These investigations showed that modification by using a PDMS stamp makes it possible to produce two-component ultrathin silane layers on Ti-containing carbon substrates. Two-component organosilane layers had higher hydrophobicity, a lower friction coefficient, and a smaller width of wear tracks than the one-component analogs. It was also found that the work of adhesion of the created surfaces had a significant influence on the value of the friction coefficient and the percentage value of the growth inhibition of bacteria.

## 1. Introduction

The widespread miniaturization of electronic devices, the introduction of new biochemical analysis techniques, and developments in medicine, modern textile production, and information technology require materials and systems with micro- or nanometer dimensions. The consequence of this trend is the development of new modification and production methods for micro- and nanomaterials and the use of modern techniques to characterize objects at the micro- and nanoscale [1,2,3,4]. An effect of miniaturization in industry is also the development of micromechanical systems (MEMSs). MEMSs are defined as devices and systems on the scale of micrometers or nanometers and are also technologies that can be used to produce some complex structures [5]. The operation of MEMS/NEMS devices can be critically influenced by adhesion, friction, wear sliding velocity, and environmental conditions, e.g., relative humidity (RH) and temperature [6].

An interesting modern material that has enjoyed growing interest in the last several years and has been successfully used for technical, medical, and biological applications is titanium-incorporated diamond-like carbon (Ti-DLC) coatings [7,8,9,10,11]. One of the most important characteristics of these coatings is chemical inertness, which allows for their use in biomedical applications.

As is known, surfaces in the medical environment can become potential reservoirs for the transmission of bacterial infections. The appropriate composition of stable surfaces with great antimicrobial properties may reduce infections in medical facilities and can also prevent biofilm formation on biomedical implants. Approximately 64% of hospital-acquired infections worldwide are attributed to the attachment of bacteria to medical implants and devices [12,13]. Hydrophobic surfaces have attracted the attention of many researchers. The modification of Ti-DLC coatings to achieve higher antimicrobial activity is important for extending their applications. Specifically, hydrophobic surfaces with very low bacterial adhesion could potentially be used in the medical and food industries [14]. In our previous work, we showed that Ti-DLC coatings (unmodified and modified with perfluoro and alkylphosphonic layers) have strong antibacterial properties [15]. It is known that TiO_2_ is characterized by good chemical stability, biocompatibility, corrosion resistance [16], and antimicrobial activity [17,18,19,20]. Moreover, DLC coatings containing TiO_2_ have good biocompatibility with osteoblasts [21]. However, the high friction coefficient and low wear resistance of this material have limited its use in the production of medical implants [7,8,9].

One of the ways to improve the tribological and physicochemical properties of Ti-DLC on the micro- and nanometer scales is to create on their surfaces one- or two-component organic monolayers that spontaneously organize on the substrate via a chemisorption process. For this purpose, organosilane compounds can be used due to the possibility of producing ultrathin films characterized by thermal and chemical resistance and good lubricating properties, the possibility of controlling the packing density and the ordering of the resulting monolayer, and moreover, the use of uncomplicated modification methods [22,23,24,25,26,27]. Furthermore, modification with organosilane compounds showed no *Pseudomonas aeruginosa* and *Desulfovibrio vulgaris* adhesion [28]. Silane-based layers showed antibacterial activity against *E. coli* and *S*. *aureus* [29]. Therefore, a system of coatings including silane self-assembled monolayers has the potential to become an excellent material that could be used in biomedical applications, for example, to cover implants or parts of the surfaces of medical devices. These surfaces would not only provide antibacterial protection but also, due to their tribological properties, prevent the excessive wear of sensitive elements of biomedical devices.

In this work, we examined the physicochemical, tribological, and antimicrobial properties of one-component (*n*-decylotrichlorosilane—DTS; *n*-dodecyltrichlorosilane—DDTS; *n*-oktadecyltrichlorosilane—ODTS; *n*-phenyltrichlorosilane—PTCS; 1H,1H,2H,2H-perflourodecylotrichlorosilane—FDTS) and two-component (DTS-FDTS, DDTS-FDTS, ODTS-FDTS, PTCS-FDTS) organosilane self-assembled monolayers on a Ti-DLC substrate. The antibacterial potential of the produced surfaces was tested against reference Gram-positive (*Staphylococcus aureus* ATCC 6538, *Staphylococcus epidermidis* ATCC 12228) and Gram-negative (*Escherichia coli* ATCC 25992, *Pseudomonas aeruginosa* ATCC 27853) bacteria, which are the cause of the greatest number of infections in medical facilities and initiate peri-implant infections.

## 2. Materials and Methods

Organosilane self-assembled monolayers were created on Ti-DLC coatings prepared on Si substrates using the radio-frequency plasma-enhanced chemical vapor deposition (RF PECVD) method with the deposition parameters described in detail in previous works [10,11]. The produced Ti-DLC coatings contained about 9.4 at.% of titanium, and the hardness and Young’s modulus were, respectively, equal to 5.5 ± 0.3 GPa and 94.1 ± 5.5 GPa. The thickness of the coating was 100 ± 2 nm. The organosilane compounds used for modification (1H, 1H, 2H, 2H-perfluorodecyltrichlorosilane—FDTS; *n*-decyltrichlorosilane—DTS; *n*-dodecyltrichlorosilane—DDTS; *n*-oktadecyltrichlorosilane—ODTS; *n*-phenyltrichlorosilane—PTCS) were purchased from ABCR, GmbH & Co., KG, Karlsruhe, Germany. These compounds were selected for the modification to determine how the type of carbon skeleton, the length of the alkyl chain, the content of fluorine atoms in the structure, and the type of terminal group affect the hydrophobic and tribological properties of the resulting SAM layers.

**Figure 1:**
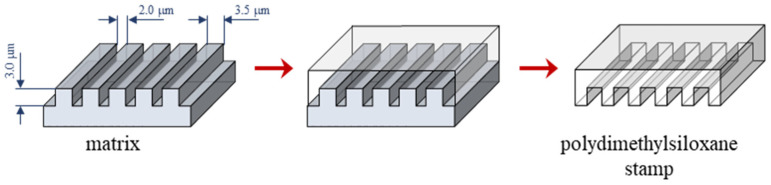
Scheme of the PDMS stamp preparation procedure.

Silicon stamps used for modification with two-component organosilane monolayers were created using the soft lithography technique combined with electron-beam lithography and the reactive-ion etching technique [30,31,32,33,34]. Si wafers with the dimensions 10 mm × 10 mm, using a 20 keV electron beam, were etched with 2 µm wide channels arranged every 2.5 µm. In the next step, polydimethylsiloxane (PDMS) resin (Silastic T2 Base Resin) and a curing agent (Silastic T2 Curing Agent), which were purchased from CH Erbslöh, Eindhoven, Netherlands were mixed at a 10:1 mass ratio. This mixture was evenly poured onto the micropatterned Si wafers and placed in a vacuum desiccator to remove bubbles. Polymer condensation on the matrix took place for 48 h at room temperature, and then the prepared PDMS stamps were mechanically removed from the matrix (Figure 1).

For the modification of Ti-DLC surfaces with one- and two-component organosilane films, the vapor phase deposition method (VPD) was used. Due to the ability of the Si-Cl bond in chlorosilanes to undergo hydrolysis, the modification process took place under reduced pressure. In the first step [33], Ti-DLC substrates were activated by low-temperature plasma for 15 min. Then, the hydrolyzed surfaces were placed into a reaction chamber and kept at low pressure (4.3 × 10^−3^ Pa). In the next step, substrates were kept in the modifier vapor for 15 min. After that, surfaces containing the adsorbed organosilane compounds were heated at 50 °C for 5 h.

**Figure 2:**
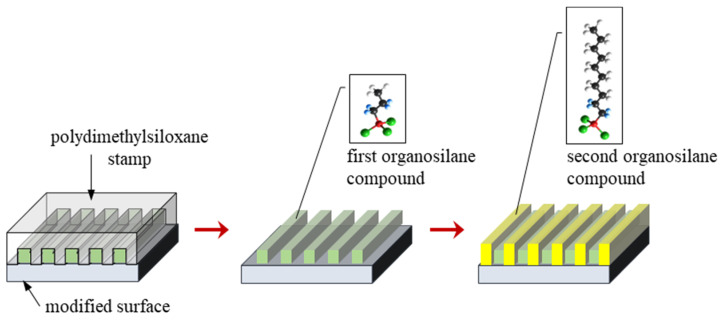
Scheme of the modification process of the surface with two organosilane compounds.

In the case of two-component layers, gas phase silanization of the modified substrates was performed two times (Figure 2). In the first step, substrates were also activated by low-temperature plasma and were covered by a PDMS stamp. Surfaces prepared in this way were placed into a reaction chamber kept under low pressure and treated with the first organosilane compound for 15 min. This compound was deposited on the Ti DLC coating through open channels made of PDMS. After the removal of the PDMS stamp, surfaces were treated with the second organosilane compound for 15 min. Finally, surfaces containing the adsorbed organosilane compounds were heated at 50 °C for 5 h. In this way, two-component organosilane monolayers on Ti-DLC surfaces for further investigations were obtained. This method was presented in our previous work [34].

### 2.1. Surface Characterization

A scanning electron microscope (Phenom G2 Pure Rotterdam, The Netherlands) was used to image the PDMS stamp surfaces for the confirmation of a successful replica of the silicon matrix substrate. The surface morphology and the root-mean-square (RMS) roughness values of two-component organosilane films were characterized using an atomic force microscopy Solver P47 apparatus (NT-MDT, Moscow, Russia) operating in the air under ambient conditions. Measurements were carried out in the tapping mode using a silicon nitride cantilever (MikroMasch, NSC/Si_3_N_4_/AlBS, Tallinn, Estonia)). The scan area was fixed at 20 µm × 20 µm.

The hydrophobicity of the modified substrates was determined by taking static and dynamic contact angle measurements using deionized water with the KRÜSS DSA25 goniometer (KRUSS GmbH, Hamburg Germany). working at ambient humidity and temperature. All the given angles were measured at three different places on each sample. For a more precise determination of hydrophobic properties, the surface free energy (SFE) with polar and dispersion components was calculated with the van Oss–Good method [35] using two additional liquids (diiodomethane and glycerol). The work of adhesion was calculated for a more detailed description of the interaction between water drops and the modified surfaces.

The tribological properties of one- and two-component organosilane thin films were characterized using a ball-on-flat microtribometer (T-23, ITEE, Radom, Poland). Counterparts in the shape of spheres with a diameter of 5.0 ± 0.1 mm made of silicon nitride (Si_3_N_4_), steel, and zirconia were used. Measurements were made over a normal load range from 30 to 80 mN at ambient humidity. The sliding speed was set at 40 mm/s, and the traveling distance was 5 mm. The wear tracks after tribological tests were examined with a scanning electron microscope (Phenom G2 Pure).

### 2.2. Biological Test

*Staphylococcus aureus* ATCC 6538, *Staphylococcus epidermidis* ATCC 12228, *Escherichia coli* ATCC 25992, and *Pseudomonas aeruginosa* ATCC 27853 were obtained from American Type Culture Collection (ATCC) (Wesel, Germany) and were grown on Luria–Bertani (LB) medium. 

The antimicrobial activity of the silane coatings against the tested bacteria was evaluated using the Japanese Industrial Standard JIS Z 2801:2000 [36] with modifications. Bacteria were cultured on Luria–Bertani (LB) medium at 37 °C on a rotary shaker. After the incubation, the test inoculum of *S. aureus*, *S. epidermidis*, *E. coli*, or *P. aeruginosa* containing 1 × 10^5^ colony-forming units (CFU per mL) in 500-fold-diluted LB medium was prepared. Next, the bacterial suspension was applied to the tested coatings in a 1 cm × 1 cm area. Ti-DLC coatings were analyzed as a control sample. After dripping the suspension of the selected bacteria onto the coatings, each sample was covered with a sterile film. Then, the samples were incubated in a moist chamber in the dark for 24 h at 37 °C. After incubation, the samples were put into a sterile tube containing phosphate buffer and vortexed. After that, the coatings and films were removed from the tubes, and with the remaining solution, a serial dilution was performed in phosphate buffer. Out of each dilution, 100 µL of the bacterial suspension was seeded on agar plates and incubated for 24 h at 37 °C. Next, the viable cells of the tested bacteria were counted. Each type of coating or solution was tested in triplicate and analyzed individually in three independent experiments. The antibacterial activity of the tested coatings was calculated as the percentage of bacterial growth inhibition (±SD) compared to the control sample without n-decylotrichlorosilane and perfluorodecylotrichlorosilane layers. 

Cell visualization was conducted in the Laboratory of Microscopic Imaging and Specialized Biological Techniques (Faculty of Biology and Environmental Protection, University of Lodz) using a Leica TCS SP8 microscope (Leica Microsystems, Wetzlar, Germany) equipped with plan achromatic objectives (Leica) and at magnifications of 63× (oil immersion) and 100× (oil immersion). The tested bacterial cells (*S. aureus* and *S. epidermidis*) were stained using the LIVE/DEAD BacLight^TM^ Bacterial Viability Kit (Thermo Fisher Scientific, Waltham, MA, USA) according to the producer’s protocol. The bacterial cells treated and untreated with silane coatings were centrifuged at 10,000 rpm for 5 min. and washed thrice with PBS. Then, the cells were suspended in 100 µL of PBS with the addition of 1 µL of a Syto 9 and propidium iodide mix (*v*/*v*:1/1). Next, the bacterial suspension (*S. aureus*, *S. epidermidis*) was vortexed and incubated at 37 °C for 15 min. in the dark. Finally, the fluorescence of Syto 9 and propidium iodide was measured at the excitation/emission (ex/em) maxima of 480/500 nm and 490/635 nm, respectively. 

The analysis of changes in the DNA content of the bacterial cells (*S. aureus*, *S. epidermidis*, *E. coli*, *P. aeruginosa*) was performed.

The test samples were prepared in accordance with the JIS Z 2801:2000 [36] procedure (described above). After incubation, the samples were put into a sterile tube containing 2 mL of phosphate buffer and vortexed. After that, the coatings and films were removed from the tubes. The samples were centrifuged for 5 min. (10,000 rpm) at 4 °C. The supernatant was transferred to new tubes. Then, 800 μL of ice-cold 96% ethyl alcohol and 30 μL of 5 M potassium acetate were added to each sample and mixed, and the samples were then stored at −20 °C for 24 h. After 24 h of incubation, the samples were centrifuged (10,000 rpm) for 15 min. at 4 °C. The supernatant was poured off, and the pellet was washed twice with room-temperature 70% ethanol. Then, the pellet was dried for 20 min. at 37 °C. The pellet was resuspended in 10 µL of sterile deionized water. The DNA concentration [ng/μL] was measured using a NanoDrop spectrophotometer (Thermo Fisher Scientific, Waltham, MA, USA).

## 3. Results

**Figure 3:**
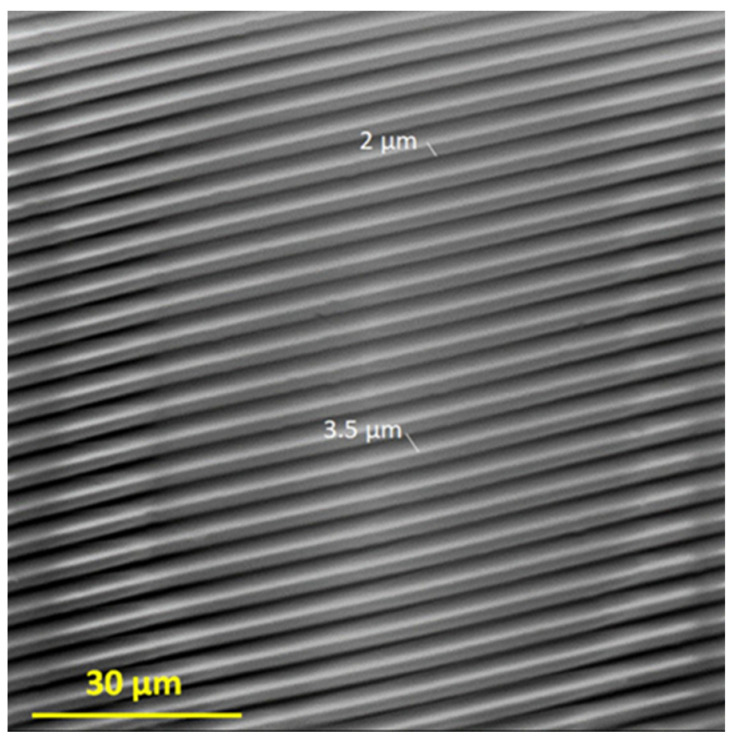
SEM image of the obtained PDMS stamp.

In this work, we used the vapor phase deposition method and PDMS stamp to prepare two-component films on the Ti-DLC substrate. The PDMS stamp, which was obtained in the first preparation step, is characterized by 2 µm wide channels arranged every 3.5 µm, the same as the output silicon matrix, which was confirmed by using scanning electron microscopy (Figure 3).

The vapor phase deposition method with a silicon stamp can be used for the preparation of thin films that have a selected pattern at the micrometer scale. This method is distinguished by the uncomplicated procedure for carrying out modifications; the microcontact printing technique has a limitation in that silicon stamps can be easily mechanically destroyed during the application or removal procedure. When this occurs, the structure of the printed film can be different from that of the initial matrix. The quality of the microcontact printing process decreases. To avoid this problem, in our studies, every single PDMS stamp was used only one time, which allowed us to perform the printing process successfully.

**Figure 4:**
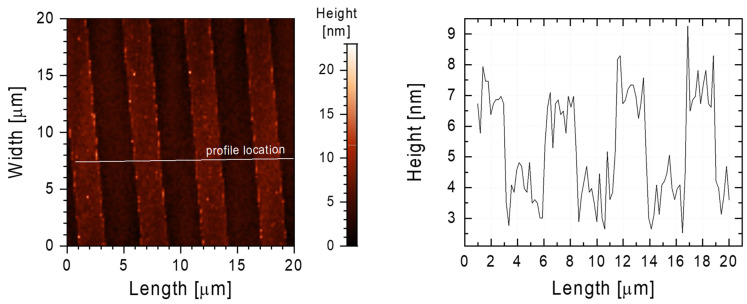
AFM image (**left**) of the FDTS-ODTS layer on the Ti-DLC surface. The white line marks the place where the surface profile was taken, shown in the chart (**right**).

AFM studies showed that two-component SAM layers were present on Ti-DLC and had an identical structure to the PDMS stamp. As seen in Figure 4, the FDTS-ODTS monolayer was characterized by 2 µm wide channels arranged every 3.5 µm, the same as the initial PDMS stamp. The obtained AFM image revealed that the used technique gives high precision in the transferred pattern, and there are no defects in the structure of the obtained thin film. In addition, these studies showed that not only the liquid phase deposition method [37] can be used for effective patterns at the micrometer scale. Using the VPD method could eliminate the influence of such factors as the concentration of the modifying solution, solvent selection, water content in the modifying solution, and aging of the solution.

The results obtained using AFM regarding the thickness/height of the Ti-DLC surface modifications (Figure 4) are consistent with the modification assumptions and were confirmed by ellipsometric measurements. Simulations performed using HyperChem 7.5. indicated that the thicknesses of single layers of FDTS, DTS, DDTS, ODTS, and PTCS were 1.64, 1.56, 1.87, 2.81, and 0.7 nm, respectively. The thicknesses of the layers determined from the ellipsometric measurements on the Ti-DLC coating modified with different compounds are comparable to the values obtained by the theoretical model determined using HyperChem 7.5. For FDTS, DTS, DDTS, ODTS, and PTCS, the values were found to be 1.95, 1.7, 2.1, 3.2, and 0.73 nm, respectively.

In order to check the effectiveness of the modification process, static measurements of the water contact angle and calculations of the surface free energy and the work of adhesion were performed (Table 1).

Based on the measurements, it was found that all surfaces modified with self-assembled monolayers were more hydrophobic than the Ti-DLC coating (Table 1). In the case of one-component SAM layers, the substrate modified by a long-chain fluorinated compound (FDTS) exhibited the highest water contact angle value (~96°). Among alkylsilane monolayers (DTS, DDTS, and ODTS), the best hydrophobic characteristics were found for the surface modified with silane with the longest carbon chain (ODTS). The presence of an aromatic ring decreased the wettability of the modified substrates to an angle value of 79°. It can therefore be concluded that modification significantly increased the value of the water contact angle and decreased the surface free energy, indicating that the surface hydrophobicity was improved compared to unmodified Ti-DLC. As a consequence, the surfaces after modification show lower work of adhesion [15,38]. Modification with two silane compounds allowed us to obtain surfaces that had higher hydrophobicity than the one-component analogs. The highest angle values were observed in two-component layers in the case of ODTS-FDTS.

For a more accurate determination of the wettability of the modified substrates, surface free energy, measured using the van Oss-Good method [35], was divided into two components: dispersion and polar (Table 1). The polar component was responsible for polar interactions between the modified surfaces and the used liquids. In the case of unmodified Ti-DLC, the contribution of the polar component was much higher (22.2 ± 1.1 mJ/m^2^) compared to that of the modified substrates. This is due to the fact that the unmodified activated coatings had hydroxyl (-OH) groups on their surfaces and thus became electron donors (Lewis bases). For Ti-DLC modified with one and two silane compounds, the values of the dispersion component were much higher than those of the polar component. The modifiers that were used in this study had nonpolar terminal groups (methyl -CH_3_, phenyl -C_6_H_5_, and trifluoromethyl -CF_3_ groups). As a result, long-range interactions between the analyzed surfaces and the used liquids (polar water, glycerin, and nonpolar diiodomethane) had a bigger contribution than polar interactions.

The next factor that helped describe the wettability of the obtained monolayers was the work of adhesion (Table 1). As is well known, the work of adhesion shows the strength of the contact between two adjacent phases (in our case, liquid and solid phases) and is related to the hydrophobic properties of the investigated surfaces. Using the Young–Dupré equation, the work of adhesion (*W_ls_*) was calculated [39]:

*W_ls_* = *γ_l_*(1 + *cosθ*)

For the liquid–solid-phase boundary, these values (Table 1) depended on the surface tension coefficient of liquid *γ_l_* (in our studies, it was water, and this value is equal to 72.8 mJ/m^2^) and the water contact angle values for the investigated surfaces [40,41,42]. A lower work of adhesion was characteristic of substrates with higher hydrophobicity (Table 1). This was due to the fact that the separation of two media (water and solid-state surface) is easier when the surface exhibits hydrophobic behavior. 

The main factor that influences hydrophobic properties is probably the structure of the two-component thin films. The difference in height between the strips is about 2 nm (Figure 4). As in the case of the Cassie–Baxter wetting model [43], a rough structure at the nanoscale reduced the surface free energy of the investigated surfaces. 

Static water contact angle measurements, surface free energy, and work of adhesion calculations allowed us to determine only the static wettability of the investigated surfaces. For further applications, interactions between the modified substrates and moving water drops are more helpful. The dynamic wettability of the modified substrates was investigated by using contact angle hysteresis measurements (Table 2). For these tests, a water drop was placed on a horizontally oriented surface. First, the volume of this drop was gradually increased (the water contact angle value was only increased to a certain limit, called the *advancing contact angle*). In the second step, the volume of the water drop was gradually decreased (the water contact angle value was only decreased to a certain limit, called the *receding contact angle*). The difference between advancing and receding contact angles is called *contact angle hysteresis (CAH)*.

Lower values of contact angle hysteresis showed that the modified substrates had higher hydrophobicity. In the case of one-component films, the lowest value of CAH was obtained for the coating modified with FDTS (12.9°). Ti-DLC coatings that were modified with two silane compounds had higher hydrophobicity (lower values of contact angle hysteresis) than the one-component equivalents.

All these measurements show that the modification of Ti-DLC with silanes increases the hydrophobicity of this material. Two-step silanization processes provided surfaces with higher water contact angle values, lower surface free energy, lower work of adhesion, and lower contact angle hysteresis. In the case of two-component organosilane thin films, coatings that contained two alkylsilanes (DTS-FDTS, DDTS-FDTS, ODTS-FDTS) had higher hydrophobicity than coatings modified with an alkylsilane/arylsilane pair (PTCS-FDTS). 

**Figure 5:**
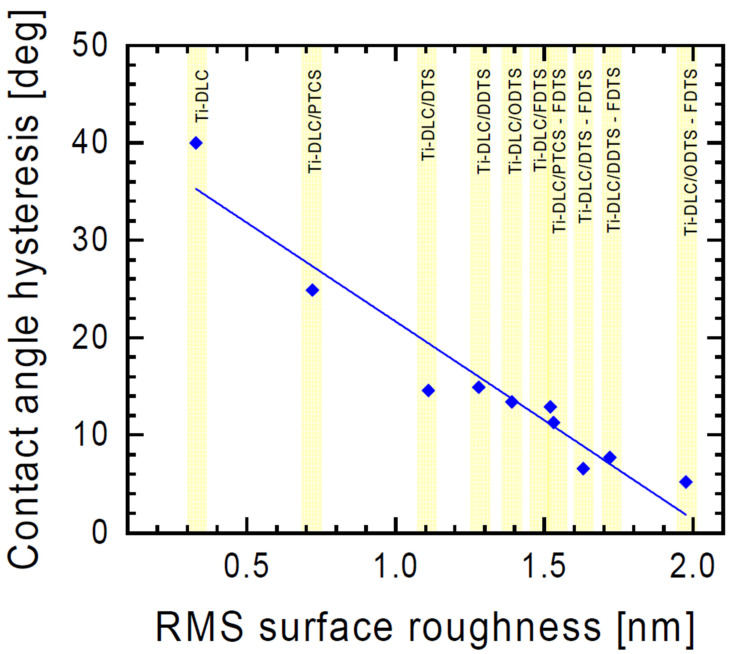
Contact angle hysteresis as a function of surface roughness of organosilane compound layers.

Hysteresis depends on many factors, such as surface roughness and inhomogeneity [44]. Our study showed that the change in hysteresis values was dependent on the surface roughness (Figure 5). After the surface modification, the hysteresis significantly decreased, indicating that the surface hydrophobicity was improved. 

In the case of mixed films, ODTS–FDTS especially has a lower surface free energy (Table 1) due to the presence of fluorine atoms in the chain and a long carbon chain length. This fact causes van der Waals attractive forces and, as a consequence, leads to lower gauche defects and the tight packing of the molecules [37].

**Figure 6:**
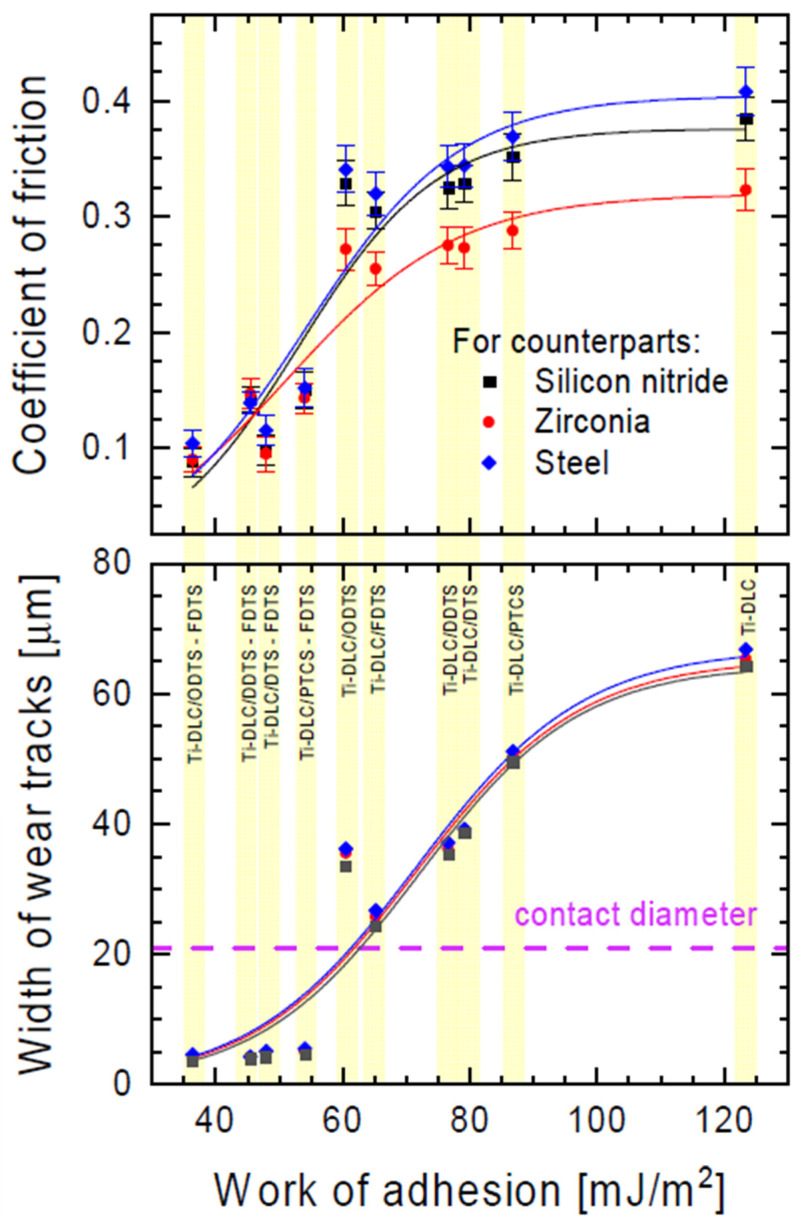
Coefficient of friction and width of wear tracks for various counterparts as a function of work of adhesion of the organosilane compound layers (see Table 1). The dashed line indicates the estimated contact diameters for the concentrated contact between the balls of the counterpart and the Ti-DLC coating for the loads applied during the friction test.

In our work, the impact of the used modifiers and their combinations on the tribological properties of the obtained monolayers was also tested. Figure 6 shows the dependence of the friction coefficient and width of the wear track as a function of adhesion work. This dependence can be described by the DoseResp function from the family of so-called growth functions. The presented graph indicates the good fit of this function to the measurement data (R^2^ > 0.9). Taking the inflection point of the obtained curve as a measure of the transition from a low to a higher friction coefficient and from a narrower to a wider wear trace, we see that these phenomena do not occur in parallel. The increase in the friction coefficient precedes the increase in the width of the wear trace by approximately 20 mJ/m^2^ of adhesion work (see Figure 6).

For one-component organosilane monolayers, the lowest friction was exhibited by the surface modified with fluorine-containing silane (FDTS). The coefficient of friction for this coating was reduced by 21% in comparison with unmodified activated Ti-DLC. In the case of non-fluorine alkylsilanes (DTS, DDTS, and ODTS), these values were reduced by 15%, and for the PTCS coating, it was reduced by only 10%. This trend is observed for all counterparts analyzed.

The tribological properties of the obtained monolayers were related to their hydrophobic characteristics. Increasing the hydrophobicity of the modified substrates and, consequently, reducing the surface free energy (in particular, reducing the dispersion component) caused lower coefficient-of-friction values. This was due to the reduction in capillary forces in the friction contact. As is well known, capillary forces (*F_k_*) depend on the tip radius of curvature *R* (in our studies, it was the counterpart) and the surface tension coefficient of liquid *γ_l_* (in our case, it was water):

*F_k_* = 4*πRγ_l_*

For this reason, the reduction in capillary forces is possible by obtaining surfaces with hydrophobic behavior. In this case, a water layer will not be created on the investigated substrates. Low capillary forces also reduce the adhesive component of friction force at the microscale. The value of friction force on a microscale can be divided into two components: the adhesive component resulting from the shear fracture of adhesive contacts between friction elements and the wear component resulting from the wear of these elements [44,45].

In addition to capillary forces, van der Waals forces also influence the adhesive component. These forces depend on the structure of the compounds that form SAM layers (primarily depending on the length of the alkyl chain and the structure of the head and terminal group). In our studies, all organosilane modifiers had the same head group (-Cl_3_), and its influence will not be described in further investigations.

The length of the carbon chain determines the ordering and the packing density of the resulting monolayer. These parameters have a significant impact on the tribological behavior of the modified surfaces. The higher packing density and more ordered SAM layers exhibited by surfaces consisting of organosilane compounds with alkyl chains of at least eight carbon atoms are the result of stronger van der Waals interactions between silane molecules [1,46]. 

Higher coefficients of friction for self-assembled monolayers consisting of short-chain alkylsilanes or arylosilanes (e.g., PTCS) can be explained by the fact that, during the formation of such SAMs, horizontal polymerization occurs at the same time as vertical polymerization (oligomerization). Vertical polymerization causes oligomers to be formed, and the obtained layers are non-ordered [47].

In the case of fluoroalkylsilanes, better tribological properties (lower friction coefficient and smaller width of wear tracks) are the result of a higher packing density of the outer part of such layers. The fluorine atoms in the terminal trifluoromethyl group of FDTS are bigger than the hydrogen atoms in the methyl groups of DTS, DDTS, and ODTS [48]. 

For mixed films, the perpendicular direction of the movement of counterparts relative to the pattern was chosen. In this case, the contact radius of the moving counterpart relative to the investigated surface changed periodically [40]. For two-component organosilane monolayers, the coefficients of friction were reduced from 45% (PTCS-FDTS) to 77% (ODTS-FDTS) in comparison to unmodified activated Ti-DLC.

**Figure 7:**
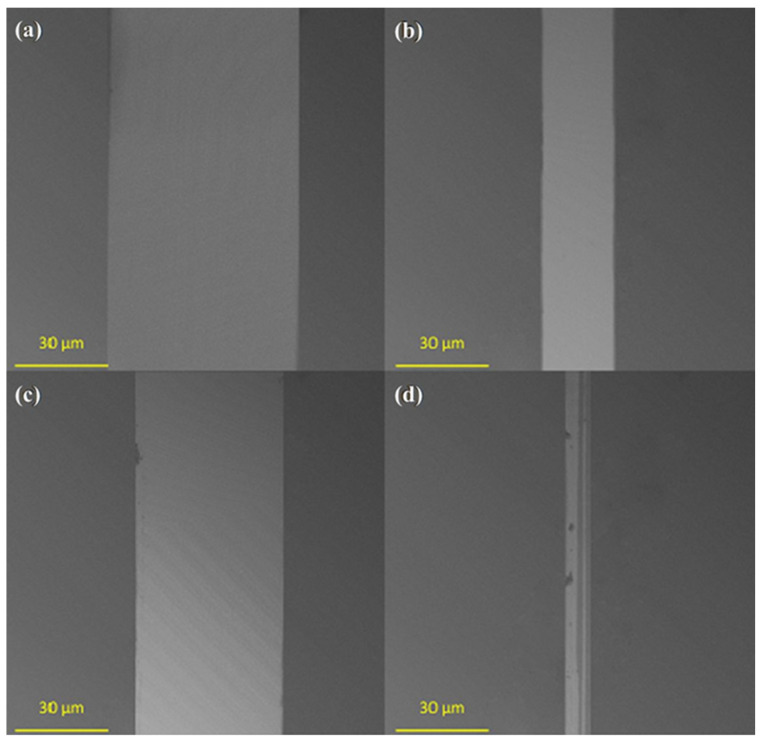
Typical SEM images of the wear tracks of (**a**) unmodified Ti-DLC, (**b**) FDTS/Ti-DLC, (**c**) PTCS/Ti-DLC, and (**d**) ODTS-FDTS/Ti-DLC.

The wear resistance of the obtained monolayers was investigated by measuring the width of wear tracks after tribological tests (Figure 6). The lowest wear resistance (Figure 7a) was observed for unmodified activated Ti-DLC (width of wear track: 64.3 µm). Modification with one-component silane monolayers (Figure 7b,c) reduced the wear of the obtained surfaces (from 49.5 µm for PTCS to 24.4 µm for FDTS). The lowest coefficient of friction (for one-component thin films, the lowest coefficient of friction was for the FDTS layer) allowed lower wear for modified substrates (for FDTS coating, approximately 24.4 µm). In the case of two-component organosilane monolayers, the highest wear resistance was for ODTS-FDTS (Figure 7d). The summary friction results for the three counterparts presented in Figure 6 prove that the presence of two-component organosilane monolayers on the Ti-DLC substrate allows for obtaining a wear trace width smaller than the estimated (Hertz formula) contact diameter for the concentrated contact between the counterpart balls and the Ti-DLC coating for the loads used during the friction test.

The antibacterial activity of the two-component self-assembled layers based on organosilane compounds was examined. The antimicrobial potential was tested against *Staphylococcus aureus* ATCC 6538, *Staphylococcus epidermidis* ATCC 12228, *Escherichia coli* ATCC 25992, and *Pseudomonas aeruginosa* ATCC 27853 using the Japanese Industrial Standard JIS Z 2801:2000.

**Figure 8:**
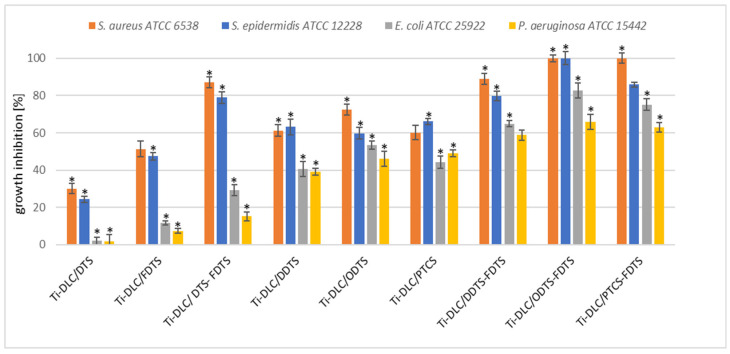
Antibacterial activity of the resulting coatings; inhibition (%) of *S. aureus*, *S. epidermidis*, *E. coli*, and *P. aeruginosa* growth after 24 h incubation. The obtained results are expressed as the mean ± SD. * represents statistically significant results (*p* ≤ 0.05) determined via one-way analysis of variance (ANOVA).

The analysis of the two-component self-assembled layers based on silane compounds revealed their antimicrobial properties against Gram-positive *S. aureus* and *S. epidermidis* strains (Figure 8). Interestingly, the Ti-DLC/DTS-FDTS and Ti-DLC/DDTS-FDTS coatings had excellent activity against *S. aureus* (90% growth inhibition compared to control). In the case of Ti-DLC/ODTS-FDTS and Ti-DLC/PTCS-FDTS, bactericidal activity against *S. aureus* was noted. The same effect was observed with Ti-DLC/ODTS-FDTS on *S. epidermidis*. Ti-DLC/DTS coatings inhibited *S. aureus* and *S. epidermidis* growth by 30% and 23%, respectively, and showed the least activity against Gram-positive strains. Gram-negative bacteria were less sensitive to the tested silane coatings. Ti-DLC/DTS and Ti-DLC/FDTS surfaces were not active against the tested Gram-negative strains. The addition of fluoride (FDTS) increased the antibacterial effect of the coating relative to Gram-positive bacteria, but in this case, the growth inhibition of *E. coli* and *P. aeruginosa* was only 10%. Gram-negative bacteria were most sensitive to Ti-DLC/DDTS-FDTS, Ti-DLC/ODTS-FDTS, and Ti-DLC/PTCS-FDTS. The growth inhibition was approximately 60–80% and 60% for *E. coli* and *P. aeruginosa*, respectively. The antimicrobial activity of the tested coatings has not been described in the literature yet. There are works confirming the antimicrobial activity of quaternary ammonium silane (QAS) compounds. Li et al. [49] characterized antimicrobial coatings fabricated with QAS copolymers. They showed effective antimicrobial activity against bacteria (*S. aureus*, *E. coli*) and a fungus (*C. albicans*). Oosterhof et al. [50] used QAS to coat silicone rubber tracheoesophageal shunt prostheses. After incubation in a throat model, all coated prostheses showed antimicrobial activity against a combination of bacteria *Staphylococcus aureus* GB 2/1, *Staphylococcus epidermidis* GB 9/6, *Streptococcus salivarius* GB 24/9, and *Rothia dentocariosa* GBJ 52/2B (36% growth reduction) and yeast *Candida tropicalis* GB 9/9 and *Candida albicans* GBJ 13/4A (16% growth reduction). Also, the organosilane quaternary ammonium compound coating on silicon rubber reduced *S. aureus* biofilm in vivo [51]. Daood et al. [52] revealed that QAS has antibacterial activity against caries-forming bacteria (*Streptococcus mutans, Lactobacillus acidophilus*). Moreover, a two-step silane coating incorporating quaternary ammonium silane resulted in a decrease in the number of active sulfate-reducing bacteria cells attached to the coated surface [53].

**Figure 9:**
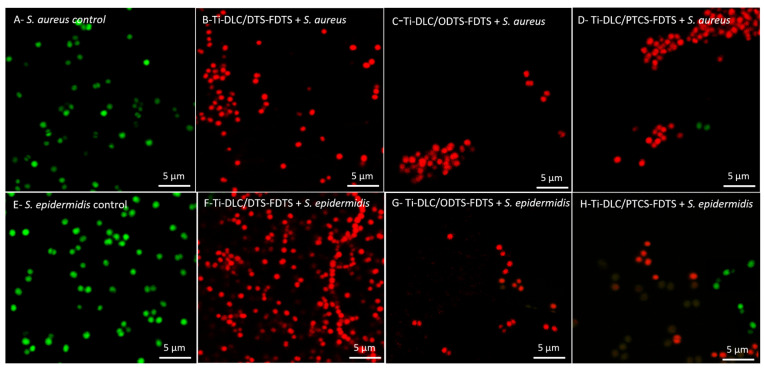
Permeability of *S. aureus* (**A**–**D**) and *S. epidermidis* (**E**–**H**) cell membranes after treatment with the tested coatings. (**A**,**E**) Control, (**B**,**F**) Ti-DLC/DTS-FDTS, (**C**,**G**) Ti-DLC/ODTS-FDTS, (**D**,**H**) Ti-DLC/PTCS-FDTS.

The influence of Ti-DLC/DTS-FDTS, Ti-DLC/ODTS-FDTS, and DLC/PTCS-FDTS coatings on the permeability of the bacterial cell membrane (for *S. aureus*, *S. epidermidis)* was also examined. The tested samples that had the best antibacterial activity against *S. aureus and S. epidermidis* were stained using the LIVE/DEAD BacLight^TM^ Bacterial Viability Kit according to the producer’s protocol. Syto 9 penetrates cells with both damaged and intact membranes, while PI penetrates only the cells with a damaged membrane and reduces the Syto 9 dye. The bacterial cells with intact membranes were stained green (Figure 9A,E), while the dead or injured cells were stained red (Figure 9B–D,F–H).

**Figure 10:**
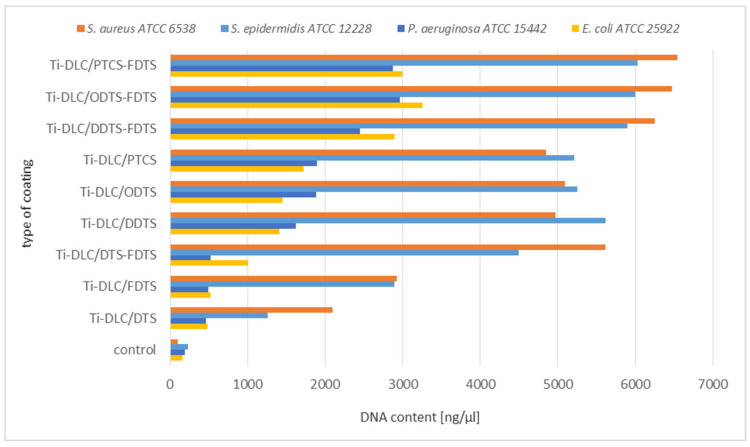
Analysis of leakage of DNA from bacterial cells incubated with two-component self-assembled layers based on organosilicon compounds.

The mechanism of action of the discussed coatings is not fully understood and requires further research. However, it can be assumed that Ti-DLC/DTS-FDTS, Ti-DLC/ODTS-FDTS, and Ti-DLC/PTCS-FDTS coatings affect bacterial membranes. This part of our research is consistent with previous results (Figure 10) and confirms the strong antibacterial activity of the tested coatings because all microorganisms in each microscope field were stained red by PI (Figure 9B–D,F–H).

The next stage of biological tests included the analysis of DNA leakage from bacterial cells incubated with two-component self-assembled layers based on organosilicon compounds.

**Figure 11:**
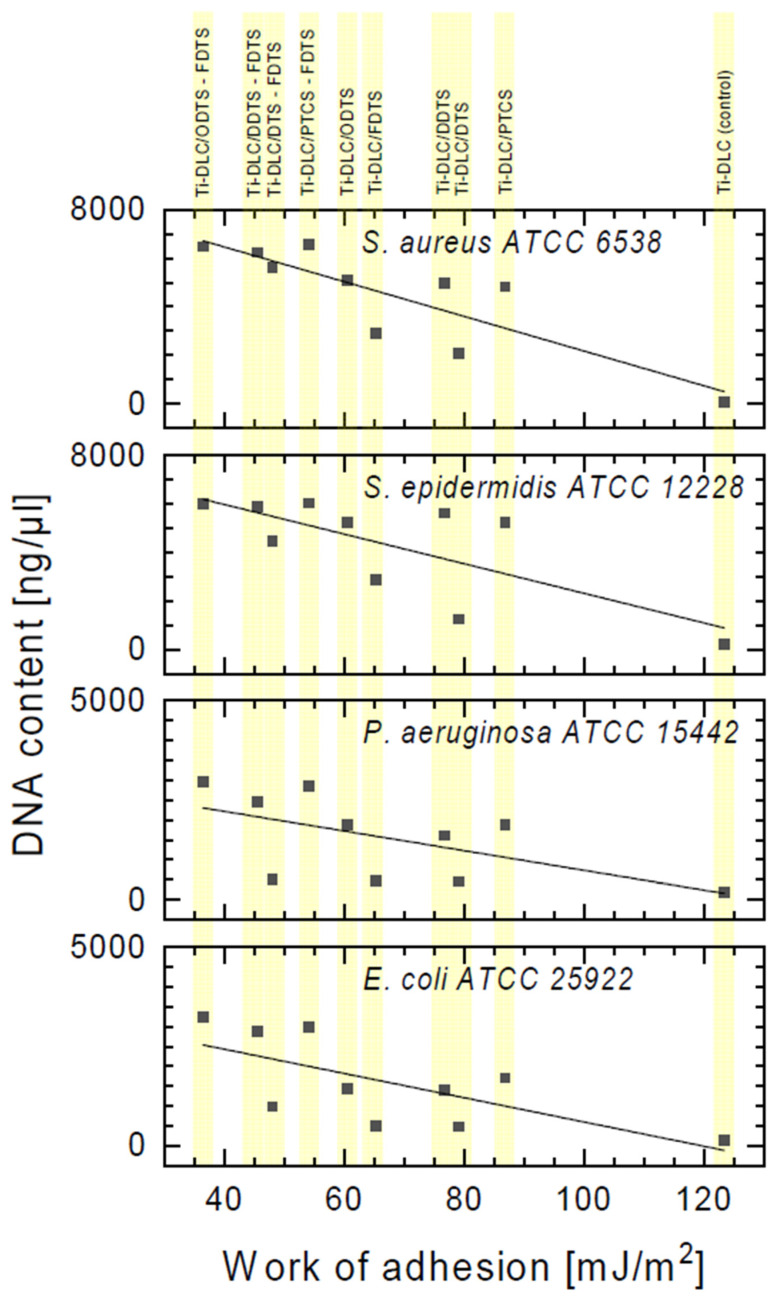
The correlation between the leakage of DNA from bacterial cells incubated with two-component self-assembled layers based on organosilicon compounds and the work of adhesion of the tested coatings.

The obtained results confirm damage to the bacterial cell membranes. Based on the above results, the significant leakage of DNA from *S. aureus* and *S. epidermidis* cells was observed in relation to the control (Figure 10). Lower leakage of DNA was observed in the case of Gram-positive strains incubated on the Ti-DLC/DTS-FDTS, Ti-DLC/DDTS, Ti-DLC/ODTS, and Ti-DLC/PTCS coatings in comparison to two-component layers (Ti-DLC/DDTS-FDTS, Ti-DLC/ODTS-FDTS and Ti-DLC/PTCS-FDTS). This part of the study confirmed the lowest antibacterial effect of the tested surfaces on Gram-negative bacteria cells, which is compatible with earlier results. The obtained result allows us to conclude that the amount of released genetic material depends on the type of surfaces, which, in combination, have a greater effect on the integrity of the cell membranes of the tested strain. Figure 11 shows the correlation between the work of adhesion of the tested surfaces and the amount of leakage of DNA from bacterial cells. An increased DNA outflow from bacterial cells was observed for surfaces with a lower work of adhesion (hydrophobic surfaces—see Table 1). 

The adhesion and subsequent growth of bacteria on surfaces are major problems in many biomedical applications. Moreover, microbial adhesion to biomaterials may also lead to implant-associated infections [54]. Bacteria in biofilms are more resistant to disinfection by a magnitude of up to 1000 times compared to free-floating microorganisms [55]. Therefore, it is very important to inhibit the growth of bacteria in the initial stage of adhesion and proliferation [56]. Silane layers could be an excellent material that may be used in biomedical applications, for example, to cover implants or parts of the surface of medical devices. The tested surfaces could not only provide excellent antibacterial protection but also, due to their tribological properties, prevent the rapid wear of sensitive elements of biomedical devices. Therefore, the search for new, stable coatings with the desired antimicrobial activity is an important task in nanotechnology, material engineering, biology, and biotechnology.

## 4. Conclusions

In summary, the vapor phase deposition method with a PDMS stamp was successfully used for the modification of Ti-DLC substrates with two organosilane modifiers. Topography studies show that the VPD method can be used to selectively modify Ti-DLC with the studied organosilane compounds. Two-component organosilane films had a more hydrophobic character (e.g., wetting angle for water of 105° and 79° for Ti-DLC/PTCS-FDTS and Ti-DLC/PTCS, respectively), a lower friction coefficient (e.g., 0.15 and 0.35 for Ti-DLC/PTCS-FDTS and Ti-DLC/PTCS, respectively, for silicon nitride counterparts), and a smaller width of wear tracks in comparison to their one-component analogs. Modification with two silanes improved the wear resistance of Ti-DLC surfaces by producing layers with lower friction coefficients. Modification with two organosilane compounds with nonpolar terminal groups improved the hydrophobic and tribological characteristics of Ti-DLC substrates. The most successful of the modifiers was ODTS-FDTS, which decreased the friction coefficient by 77%. 

The analysis of the antimicrobial properties of self-organizing layers deposited on Ti-DLC showed the growth inhibition of both groups of bacteria tested: Gram-positive and Gram-negative. Ti-DLC/ODTS-FDTS and Ti-DLC/PTCS-FDTS coatings showed bactericidal activity against *S. aureus*, while Ti-DLC/DTS-FDTS and Ti-DLC/DDTS-FDTS caused 90% inhibition of *staphylococcal* growth. The influence of changes in the hydrophobicity of the surfaces on the bacterial growth on the studied surfaces was shown. The obtained results show that Ti-DLC coatings modified with self-assembled monolayers can be used in tribological and biomedical applications.

## Figures and Tables

**Table 1:** Water contact angle values, surface free energy with dispersion and polar components, and work of adhesion obtained for one- and two-component organosilane films.

Substrate/Modifier	Water Contact Angle (deg)	Surface Free Energy (mJ/m^2^)	Dispersion Component (mJ/m^2^)	Polar Component (mJ/m^2^)	Work of Adhesion (mJ/m^2^)
Ti-DLC	46 ± 3	68.7 ± 3.4	46.5 ± 2.3	22.2 ± 1.1	123.4
Ti-DLC/FDTS	96 ± 2	32.0 ± 1.6	31.1 ± 1.5	0.9 ± 0.1	65.19
Ti-DLC/DTS	85 ± 1	37.8 ± 1.9	34.9 ± 1.7	2.9 ± 0.2	79.1
Ti-DLC/DDTS	87 ± 2	35.5 ± 2.0	32.8 ± 1,8	2.7 ± 0.2	76.6
Ti-DLC/ODTS	95 ± 2	33.7 ± 1.7	31.4 ± 1.6	2.3 ± 0.1	60.5
Ti-DLC/PTCS	79 ± 2	42.9 ± 2.2	39.1 ± 2.0	3.8 ± 0.2	86.7
Ti-DLC/DTS-FDTS	110 ± 2	16.9 ± 1.1	16.3 ± 1.0	0.6 ± 0.1	47.9
Ti-DLC/DDTS-FDTS	112 ± 2	14.2 ± 1.1	13.8 ± 1.0	0.4 ± 0.1	45.5
Ti-DLC/ODTS-FDTS	120 ± 2	12.5 ± 1.0	12.3 ± 0.8	0.2 ± 0.2	36.4
Ti-DLC/PTCS-FDTS	105 ± 2	24.6 ± 1.1	22.2 ± 0.9	2.2 ± 0.2	54.0

**Table 2:** Advancing and receding contact angle values and contact angle hysteresis obtained for one- and two-component organosilane films.

Substrate/Modifier	Advancing Contact Angle (deg)	Receding Contact Angle (deg)	Contact Angle Hysteresis (deg)
Ti-DLC	67.2	27.2	40.0
Ti-DLC/FDTS	103.8	90.9	12.9
Ti-DLC/DTS	95.7	81.1	14.6
Ti-DLC/DDTS	98.0	83.1	14.9
Ti-DLC/ODTS	103.2	89.8	13.4
Ti-DLC/PTCS	92.5	67.6	24.9
Ti-DLC/DTS-FDTS	115.8	109.2	6.6
Ti-DLC/DDTS-FDTS	117.9	110.2	7.7
Ti-DLC/ODTS-FDTS	125.8	120.6	5.2
Ti-DLC/PTCS-FDTS	108.5	97.2	11.3

## Data Availability

Data are contained within the article.
